# An extremely rare case of malignant jejunal mesenteric inflammatory myofibroblastic tumor in a 61-year-old male patient: A case report and literature review

**DOI:** 10.3389/fmed.2022.1042262

**Published:** 2022-11-08

**Authors:** Hamdi Al Shenawi, Salamah A. Al-Shaibani, Suhair K. Al Saad, Fedaa Al-Sindi, Khalid Al-Sindi, Noor Al Shenawi, Yahya Naguib, Rami Yaghan

**Affiliations:** ^1^Department of Surgery, College of Medicine and Medical Sciences, Arabian Gulf University, Manama, Bahrain; ^2^College of Medicine and Medical Sciences, Arabian Gulf University, Manama, Bahrain; ^3^Department of Pathology, King Hamad University Hospital, Busaiteen, Bahrain; ^4^Department of Physiology, College of Medicine and Medical Sciences, Arabian Gulf University, Manama, Bahrain; ^5^Department of Clinical Physiology, Faculty of Medicine, Menoufia University, Menoufia, Egypt; ^6^Department of Surgery, Jordan University of Science and Technology, Irbid, Jordan

**Keywords:** inflammatory, myofibroblastic, stromal, mesenchymal, tumor, jejunum, mesentery, mass

## Abstract

**Introduction:**

A mesenteric inflammatory myofibroblastic tumor (IMT) is a rare solid tumor of intermediate malignant potential that affects children, adolescents, and young adults predominantly. IMT is mostly encountered in the lung. We report a case of malignant jejunal mesenteric IMT in a 61-year-old male patient who presented with vague abdominal pain and generalized weakness. CT scan revealed a mesenteric mass displacing the attached jejunum. Surgical resection was curative.

**Discussion:**

An extensive literature review was performed to update and further analyze the already available data. A total of 35 cases with mesenteric IMT were reported previously. Only five cases of jejunal mesenteric IMT were reported. Mesenteric IMT demands vast effort to reveal the diagnosis due to its vagueness in the clinical presentation. Mesenteric IMT resembles each other in plenty of pathological and immunohistochemical characteristics.

**Conclusion:**

To the best of our knowledge, this is the first case of malignant jejunal mesenteric IMT in the elderly. Surgical resection was curative.

## Introduction

An inflammatory myofibroblastic tumor (IMT) of the lung was first reported in 1939 by Brunn (1). Classically, IMT was known for its benign propensity and local involvement. Locally malignant variants (with metastases seldomly) were later described. Currently, the World Health Organization states its nature as an intermediate malignant neoplasm with mesenchymal origin (2). IMT has been reported in almost all body and soft tissue visceral organs (3, 4). Most recorded cases were in the lung, followed by the mesentery and the omentum (5, 6). IMT has also been known as inflammatory pseudotumor, plasma cell granuloma, omental mesenteric myxoid hamartoma, and inflammatory fibrosarcoma. This reflects the diversity in the clinicopathological pictures of this tumor (7). The etiology of IMT remains unclear. However, it has been significantly related to viral infection (HHV-8), trauma, and surgery (8). The anaplastic lymphoma kinase (ALK) gene exists in 33–67% of IMT cases, with a higher prevalence in children and young adults (9). Microscopically, IMT is composed of spindle-shaped cells of myofibroblasts accompanied by inflammatory cells (10). There are no distinctive signs or symptoms for IMT, due to its ability to affect various anatomical sites (11). Herein, we report a rare case of malignant IMT in the jejunal mesentery of a 61-year-old male patient who presented with abdominal pain and generalized weakness.

## Case report

**FIGURE 1:**
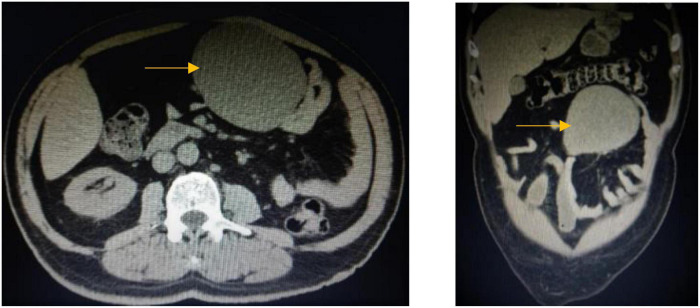
CT scan of the abdomen, coronal, and axial images shows large well-defined opacity at mesentery on the left side of the upper abdomen surrounded by small and large bowel loops with no enlarged mesenteric or retroperitoneal lymph nodes.

**FIGURE 2:**
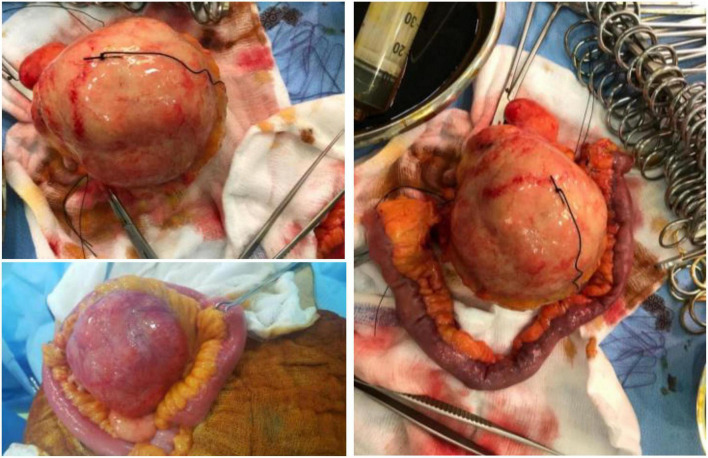
Gross image of the tumor with associated bowel loop.

A 61-year-old male patient presented with vague progressive abdominal pain and generalized weakness for a 6-month duration. He is married with four children. He is a non-smoker and a mechanical engineer. General clinical examination revealed paleness. Abdominal examination revealed a central intra-abdominal mass. Complete blood count (CBC), liver, and renal function tests were normal apart from a low hemoglobin (Hb; 12.15 g/dl) and a high erythrocyte sedimentation rate (ESR; 22 mm/h). Abdominal ultrasound (US) revealed a well-defined solid hypoechoic mesenteric mass measuring about 7.8 × 8 cm that was situated in the upper abdomen above the umbilicus. CT scan showed a mesenteric mass with no evidence of intestinal obstruction (Figure 1). These findings gave an ambiguous diagnosis of a solid mass in the mesentery (Figure 1). Fine-needle aspiration cytology (FNAC) or true-cut biopsy was not recommended by the radiologist due to the absence of a safe window. Exploratory laparotomy was the decisive treatment for this patient. Intraoperatively, a spheroid-like mass was occupying the mesentery of the jejunum with an intact jejunal wall (Figure 2). The whole mass was resected with partial jejunectomy followed by end-to-end anastomosis. There was no gross lymphadenopathy. Cytological evaluation of the peritoneal fluid was negative for malignant cells.

Provisional diagnosis included gastrointestinal stromal tumor (GIST) and IMT. The literature reported other differential diagnoses of mesenteric mass (12). The most common one was desmoid tumor with other possibilities such as teratoma, liposarcoma, hemangioma, neuroblastoma, carcinoid tumor, and adenocarcinoma (12).

A gross examination of the specimen revealed a large tannish glistening tumor mass in the center of the bowel loop. The rest of the bowel and the attached pericolic fat were normal (Figure 2).

**FIGURE 3:**
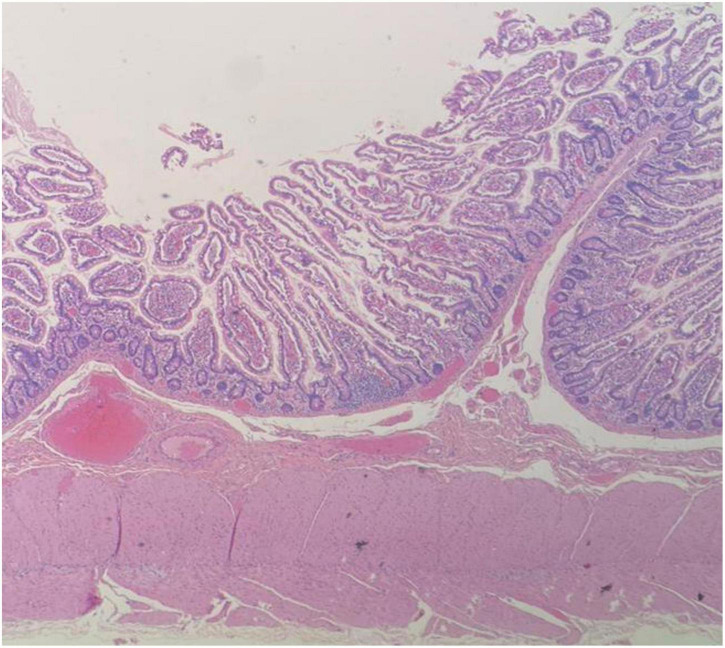
Microscopic image of intact jejunal bowel adjacent to tumor site, LPF.

**FIGURE 4:**
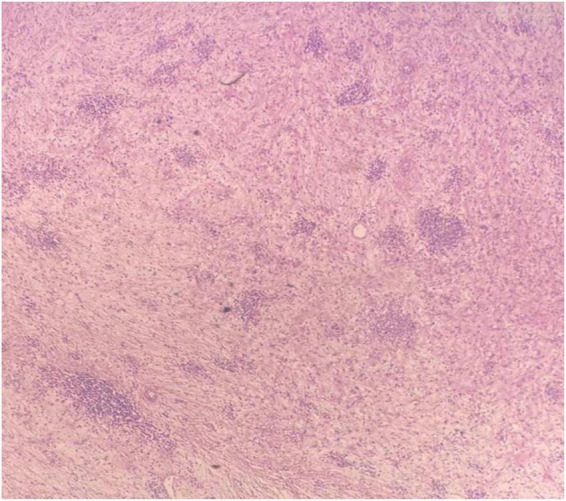
Microscopic image of loosely arranged spindle cells growing in haphazardly arranged fascicles, interspersed by abundant chronic inflammatory cells, LPF.

**FIGURE 5:**
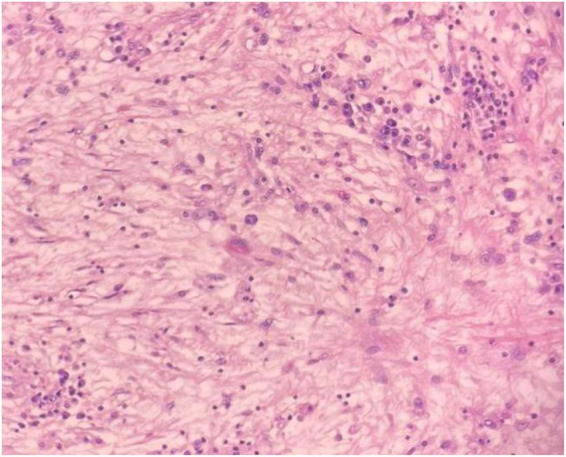
Microscopic image of abundant chronic mononuclear and inflammatory cells, mostly lymphocytes, plasma cells, and histiocytes accompanied by eosinophils and scanty lymphoid aggregations, HPF.

The pathologist described a mesenteric-origin tumor that occupied the visceral peritoneum with an intact jejunal loop (Figure 3). Microscopically, the tumor was composed of myofibroblastic spindle-shaped cells with absent mitosis and minimal atypia (Figure 4). The tumor contained abundant chronic mononuclear and inflammatory cells, mostly lymphocytes, plasma cells, and histiocytes accompanied by eosinophils. Lymphoid aggregations were scant (Figure 5). Further immunohistochemistry studies were performed by another independent pathologist: the tumor had negative immunoreaction to CD117, DOG1, desmin, and anaplastic lymphoma kinase (ALK-1). There was an infrequent immunoreaction to smooth muscle actin (SMA).

The post-operative course was uneventful, and he returned to his job within 2 weeks of the surgery. He was asymptomatic for the last four and a half years. Follow-up US of the abdomen yearly and CT scan of the abdomen every 2 years were done with no clue of tumor recurrence or mass lesion.

## Discussion

Inflammatory myofibroblastic tumor is a mesenchymal, rare solid tumor of intermediate malignant potential (2). IMT imitates malignant neoplasm, with a local recurrence rate of up to 25% (8, 13). Based on demographic data, IMT often affects children and young adults (14). While some reported female predominance in IMT cases (female:male = 1.67:1) (15), others reported the opposite sex ratio (16). A high recurrence rate is much attributed to the tumor site, pathological features (multifocal or ill-defined), and incomplete resection of the tumor. A significant correlation between IMT that arises in the abdomen, mesentery, omentum, or retroperitoneum and the aggressive local recurrence has been documented. In addition, IMT with gross multinodular features and a diameter of more than 8 cm has a more aggressive course (6, 17).

Tables 1–3 summarize 36 case reports (e.g., the present case) of mesenteric IMT, emphasizing clinical, radiological, and pathological features, respectively. The literature review was performed by searching electronic databases (PubMed and ScienceDirect) and using the search terms “Inflammatory, myofibroblastic, tumor, Jejunum, mesentery, mass.”

**TABLE 1:** Clinical features of the reported cases of inflammatory myofibroblastic tumor of the mesentery.

References #	Age/Gender	Clinical presentation	Location	Treatment	Follow up/Recurrence
**Our case**	61 years/male	Vague abdominal pain, generalized weakness, anemic and palpable abdominal mass.	Mesentery of jejunum.	Laparotomy excision of the whole mass with end-to-end anastomosis of attached bowel.	No recurrence.
(5)	11 months/female	Gradual progressive abdominal distension and large, firm lump in paraumbilical region. It was moving with respiration.	Mesentery of small bowel.	Laparotomy: excision of the mass in mesentery and involved small bowel with end-to-end anastomosis.	Recurred 6 months after first excision, 1.8 × 1.8 × 2.3 cm tumor, well-defined, heterogeneous in the right paravesical region. Another lesion in the left side, leaning on the spleen, measuring 4.4 × 2.1 × 3 cm. No infiltration into the organs. Treated with ceritinib (systemic therapy) which showed good response.
(6)	63 years/male	Abdominal pain with palpable abdominal mass in the left lower quadrant.	Mesentery of proximal jejunum.	Laparotomy: resection of 30 cm jejunum and its mesentery with end-to-end anastomosis and resection of a 0.5 cm nodular mass at the mesentery of distal ileum.	Recurred 3 months after first excision. the tumor was 19*17*10 cm, involving the mesentery of both ileum and jejunum. And invaded small bowel and sigmoid colon. It was diagnosed by CT scan. Re-excision of the mass, ileum, and jejunum with their mesentery and part of sigmoid colon with subsequent end-to end anastomosis.
(8)	7 years/male	Inability to pass gas or feces “obstipation” abdominal pain, leukocytosis, and elevated c-reactive protein.	Mesentery of ileum.	Laparotomy resection of enlarged ileal segment and the appendix.	No recurrence.
(18)	59 years/male	Abdominal pain and firm mobile periumbilical mass.	Mesentery of jejunum.	Laparotomy incomplete resection of the mass, leaving the tissues of tumor that infiltrated the root of mesentery, superior mesenteric vessel along with lower edge with the third portion of duodenum. Retroperitoneal as well.	Recurred 3 months after first excision, treated with medical therapy: prednisone for 7 days followed by indomethacin. Follow-up: no evidence of recurrence.
(19)	57 years/male	Voluminous, indurated, mobile palpable mass in right hypochondrium.	Transverse mesocolon.	Exploratory laparotomy excision of the mass completely, segmental colectomy of transverse colon and primary anastomosis.	No recurrence in 4 years.
(20)	10 months/male	Abdominal lump in abdomen.	Mesentery of small bowel.	Laparotomy resection of the mesenteric tumor with a small segment of attached bowel.	No recurrence in 4 years of follow-up.
(21)	3 years/female	Abdominal pain and distension at McBurney point, bilious vomiting, and leukocytosis.	Mesentery of jejunum.	Laparoscopy: moderate hemoperitoneum. Laparotomy resection of 25 cm of jejunum and associated mesentery with end-to-end anastomosis.	Follow up with NSAIDs and MRI scan/No recurrence.
	20 months/male	Non-bloody diarrhea, vomiting, abdominal pain with rebound tenderness, and weakness. Leukocytosis. History of trauma insult.	Small bowel mesentery.	Laparoscopy: hemoperitoneum and ecchymosis with torn peritoneum along the abdominal wall on the right side. Laparotomy: drainage of sanguinous fluid. Resection of ischemic bowel and avulsed mesentery with primary anastomosis.	Follow up with NSAIDs and MRI scan/No recurrence.
(22)	7 months/male	Abdominal distension.	Mesentery of small bowel and omentum.	Laparotomy: resection of the mass in the mesentery and multiple nodules in the omentum.	Recurred 8 months after first excision. Re-excision of 4 cm paracolic mass and several small peritoneal and mesenteric implants.
(23)	5 months/female	Abdominal mass, anemia.	Mesentery of ileum. 25 cm from the ileocecal junction.	Laparotomy resection of the mass with 6 cm of the ileum. Followed by end-to-end anastomosis.	No recurrence.
	1 year/male	Anemia, failure to thrive, and multiple palpable swellings on abdomen.	Mesentery of ileum.	Laparotomy excision of masses with 20 cm of ileum followed by end-to-end anastomosis. And restoration of gut continuity.	No recurrence.
(24)	4 months/male	Fever, abdominal distension, jaundice, and elevated C-reactive protein. Palpable fixed hard mass with prominent superficial veins in the abdomen.	Mesentery of colon.	Laparotomy: Multiple satellite lesions were observed. Excision of lymph nodes, the lesion in the supra-umbilical area, the infiltrated colon with margins, and 6 cm only of the mesenteric tumor (12*15 cm). Followed by colostomy. The patient was given fresh frozen plasma and packed red blood cells.	They planned to follow the child for 12–24 weeks with radiological investigations and biopsy.
(25)	28 years/female	Weight loss, abdominal pain, palpable periumbilical mass. She had preexisting constipation.	Small bowel mesentery.	Exploratory laparotomy with total resection of the tumor and the affected section of colon and jejunum.	ND.
(26)	34 years/male	Abdominal pain, photophobia, uveitis, fatigue, anemia, diaphoresis, weight loss, palpitation, and anemia. Past history of 2 seizures and transient ischemic attack.	Mesentery of small bowel.	Exploratory laparotomy excision of the mesenteric mass, right hemicolectomy, and resection of 15 cm of terminal ileum with lymph nodes.	1 Week post-operatively, he was diagnosed with anterior uveitis and treated with steroid drops. Resolved after 1 month. They decided to follow up the patient with yearly CT to evaluate recurrence. But no results documented.
(27)	37 years/male	Abdominal pain in the epigastric and periumbilical region, and palpable abdominal mass, fever, weight loss, weakness, and leukocytosis.	Mesentery of ileo-jejunal junction.	Laparotomy resection of 20 cm of small bowel with the mass.	No recurrence.
(28)	67 years/male.	Abdominal pain that is worse with movement, recent coryzal illness, nausea and vomiting. Past history of asthma, peripheral vascular disease, inguinal hernia, and irritable bowel syndrome. Right side peritonism. Leukocytosis, elevated CRP.	Mesentery of ileum.	Laparoscopy: blood stains in the free fluid, inflamed segment of ileum, and thickened hypervascular mesentery. Laparotomy: resection of the inflamed segment of small bowel with the mass in the mesentery and its connected grossly normal mesentery. Followed by end-to-end anastomosis.	Clinicoradiological follow-up. The results were ND.
(29)	19 years/female	Abdominal pain, nausea, and vomiting.	Mesentery of terminal ileum.	Laparotomy resection of tumor with the terminal ileum and ileocecal segment.	Recurred 9 weeks after the first excision. Clinically had acute renal failure. Radiology findings: masses in abdomen with large amount of ascitic fluid. Treated with chemotherapy but died after 3 weeks.
	39 years/male	Laborious urination, abdominal distension, and abdominal pain.	Mesangial region of sigmoid and rectum. “Sigmoid mesocolon”	Surgical excision of the tumor in the mesentery with attached sigmoid, upper rectum and left ureter. Followed by Chemotherapy.	Recurred 4 months after first excision, diagnosed with CT. And treated with chemoembolization. Died after 12 months.
(30)	14 years/female	Abdominal pain, palpable, painless, hard, and firm abdominal mass in the right side of umbilicus.	Mesentery of small bowel.	Laparotomy incomplete resection of the mass because the tumor was surrounding the superior mesenteric artery branches. Biopsy was taken.	3 Weeks after surgery, the patient received chemotherapy (methotrexate, cisplatin) and NSAIDs (diclofenac sodium). 3 years of follow up: No recurrence.
(31)	28 years/female	Intermittent abdominal pain for 2 years associated with nausea and vomiting.	Mesentery of small bowel.	Laparotomy: Complete resection of the tumor was impossible due to fibrotic nature of the tumor and adherence to the superior mesenteric artery. After 2 weeks later: patient was given prednisone, celecoxib (NSAIDs), and pantoprazole for 8 weeks	No recurrence. 3 months later: CT scan revealed near-complete resolution of the prior mesenteric inflammation and decrease in size of the mass to 3.5*4.2 cm.
(32)	8 years/ male	Anemia, culture-negative fever (pyrexia of unknown origin), night sweats, high sedimentation rate, and thrombocytosis.	Transverse mesocolon.	Laparotomy resection of the solid mass that was attached to the serosa of the transverse colon. And resection of the attached segment of colon.	ND.
(33)	32 years/male	Abdominal pain, anorexia weight loss, palpable mass, anemia, hypergammaglobulinemia, prolonged prothrombin time, high ESR.	Mesentery of jejunum.	Laparoscopic partial resection of the tumor with 21 cm of jejunum.	No recurrence.
(34)	7 years/female	Abdominal lump. Well-defined, firm, ovoid, and mobile mass on the right side of the umbilicus.	Mesentery of ileum.	Complete laparotomy excision of the mass with resection of the adherent bowel and ileo-transverse anastomosis.	No recurrence.
(35)	5 years/male	Mobile abdominal mass at the midline.	Mesentery of ileum.	Laparotomy resection of the tumor with 20 cm of attached ileocecal valve followed by end-to-end anastomosis. The mesentery was restored.	ND.
(36)	35 years/female	Abdominal lump that was solitary, spherical and smooth in the right upper abdomen. Weight loss.	Transverse mesocolon and omentum.	Laparoscopic excision of the tumor with segmental colectomy.	No recurrence.
(37)	13 years/female	Fatigue, fever, non-bloody diarrhea, painless abdominal mass, thrombocytosis, anemia, elevated ESR, and hyperglobulinemia (IgG).	Mesentery of the ileocecal region.	Laparotomy excision of the mass, terminal ileum, part of cecum. Followed by ileocecal anastomosis.	No recurrence.
	15 years/male	Low-grade fever, night sweating, weight loss, abdominal pain. painless mass in the right abdomen, thrombocytosis, anemia, elevated ESR.	Ileocecal mesentery.	First laparotomy: mass excision and biopsy. Second laparotomy after 6 days: excision of the terminal ileum and mesentery. Followed by ileocolic anastomosis.	No recurrence.
(38)	14 months/female	Intermittent abdominal pain, vomiting, palpable and tender abdominal mass, and weight loss.	Mesentery of duodenojejunal junction.	Laparotomy excision of mass with 33 cm of jejunum followed by jejuno-jejunal anastomosis.	No recurrence.
(39)	67 years/ female	Abdominal fullness on early satiety and palpable, mobile non-tender lump in the left lumbar region.	Transverse mesocolon.	Laparotomy: resection of mesenteric tumor, a segment of transverse colon and descending colon including splenic flexure.	No recurrence after 4 months of follow-up.
(40)	28 years/ female	Asthenia (lack of energy), appetite loss, abdominal pain, night swat, and fever.	Mesentery of the terminal ileum.	Laparotomy: resection of the mesenteric mass, 25 cm of terminal ileum, and 6 lymph nodes. Followed by ileocecal anastomosis.	No recurrence.
(41)	38 years/female	Painful abdominal pain, nausea, vomiting, tender and palpable mass in left hypochondrium. Past history: gastric banding 7 years earlier but removed later. Then gastric bypass during the same period.	Mesentery of proximal jejunum.	Laparotomy: they performed adhesiolysis to expose the mass. Then the tumor with six feet of infiltrated jejunum was resected. Gastric bypass was reversed.	No recurrence after 2 years of follow-up.
(42)	4 months/female	Rapid growing abdominal lump and large non-tender mass, mobile, and palpable.	Mesentery of ileum.	Laparotomy: excision of the mesenteric mass, attached ileal loops, and mesenteric lymph nodes. Followed by ileo-ileal anastomosis.	No recurrence after 6 months of follow-up.
(43)	35 years/female	Loss in body weight and solid palpable abdominal mass.	Mesentery of colon.	Laparoscopic excision of the tumor and partial colectomy.	No recurrence after 6 months.
(44)	4 months/male	Palpable, soft, and non-tender mass in the right upper quadrant.	Mesentery of ileum.	Laparotomy: excision of the mesenteric tumor with preservation of ileum.	No recurrence in 3 months of follow-up.
(45)	34 years/female	Abdominal pain in the right side.	Mesentery of ileocecum.	ND.	Lost to follow up.
(46)	73 years/female	Lower abdominal mass.	The mesentery of the small bowel.	Surgical Resection of the mesenteric tumor, right hemi colon, and terminal ileum since there was an invasion.	Three months later, Unluckily, a CT of the abdomen revealed an aggressive recurrence at the mesentery and was associated with liver metastasis.
(47)	9 years/female.	Chronic Abdominal pain, weight loss, anorexia. Pelvic mass, anemia, thrombocytosis, hypergammaglobinemia, elevated erythrocyte sedimentation rate, and high fibrinogen.	The mesentery of the distal ileum.	Surgical resection of the mesenteric mass, nearby ileum, and appendix. Biopsy taken for retroperitoneal lymph nodes.	Follow-up after 3 years showed no recurrence.
	7 years/female.	Anorexia, and daily fever. Suprapubic mass, refractory microcytic anemia, thrombocytosis, elevated ESR.	Small bowel mesentery, it was attached to the segment of mid-small bowel, rectosigmoid, posterior wall of uterus, right broad ligament, and dome of bladder.	Laparoscopic resection of the mesenteric mass that measures 8*6.5 cm at the lower abdomen.	No recurrence.
	5 years/male	Fever, failure to growth, poor appetite, and urinary frequency. Refractory Microcytic anemia, thrombocytosis, hypoalbuminemia, hyperglobinemia, high erythrocyte sedimentation rate. He had mobile, palpable abdominal mass.	Mesentery of distal ileum.	Surgical resection of the mesenteric mass, 11 cm of ileum, cecum, and mesenteric lymph nodes.	Follow-up after 3 years revealed no recurrence.
(48)	2 years/male	Intermittent Fever, failure to growth, and chronic constipation. Firm abdominal mass in RLQ. Anemia, thrombocytosis, hypergammaglobinemia, high erythrocyte sedimentation rate.	Mesentery of small intestine and measures 10*10*6 cm.	Surgical resection of a mass in the mesentery, terminal ileum and cecum followed by ilio-colic anastomosis.	5 Months after, he presented with similar presentation, CT scan revealed a mass that is located above the bladder. So, another surgical resection of 4*6 cm mass along with small portion of bladder, and another 1 cm mass in the omentum. Two years follow up- no recurrence.

**TABLE 2:** Radiological features of the reported cases of inflammatory myofibroblastic tumor of the mesentery.

References #	Age/Gender	Radiology findings	Preoperative diagnosis	Location
Our case	61 years/male	US: showed well defined hypoechoic solid abdominal mass. CT scan: showed large well defined opacity at mesentery on the left side of upper abdomen surrounded by small and large bowel loops with no enlarged mesenteric or retroperitoneal lymph nodes could be due to desmoid tumor or GIST.	Solid mesenteric mass.	Mesentery of jejunum.
(5)	11 months/female.	US: irregular heterogeneous, hypoechoic mass with vascularity. It was in the right region of abdomen, leaning on the liver superiorly and displacing right kidney posteriorly. Also, it was pushing the bowel loops to the left side. CT scan: Irregular hypodense mesenteric mass with mild heterogeneous post contrast enhancement.	Mesenteric mass.	Mesentery of small bowel.
(6)	63 years/male	CT scan: Heterogeneous mass in mesentery with central necrosis without bowel involvement nor lymph nodes. CXR: no lesion.	Intra-abdominal tumor.	Mesentery of proximal jejunum.
(8)	7 years/male.	Plain abdominal x-ray: signs that suggest small bowel intussusception. US: target sign in small bowel and signs of appendicitis.	Intestinal intussusception and appendicitis.	Mesentery of ileum.
(18)	59 years/male.	US: Homogeneous hypoechoic mass. CT scan: well-delineated intraperitoneal tumor. It had homogenous texture and a peripheral hypodensity, that suggested peripheral necrosis.	Differential diagnosis: sarcoma, adenocarcinoma, lymphoma, gastro-intestinal stromal tumor, desmoid, carcinoid tumor.	Mesentery of jejunum.
(19)	57 years/ male	CT scan: single, round, and huge mass with heterogeneous density. Calcified and thick wall. It compresses intestinal loops, the uncinate process of pancreas, and part of duodenum.	Gastrointestinal stromal tumor.	Transverse mesocolon.
(20)	10 months/male	CT scan: showed large heterogeneously enhancing mass in the mesentery.	IMT. Diagnostic by fine needle aspiration cytology (FNAC).	Mesentery of small bowel.
(21)	3 years/female	US: fluid in right hemiabdomen and debris.	Perforated appendicitis.	Mesentery of jejunum.
	20 months/male	US: complex fluid in abdomen, edematous mesentery, and thickened loops of bowel.	Perforated appendicitis.	Small bowel mesentery.
(22)	7 months/male	ND.	ND.	Mesentery of small bowel and omentum.
(23)	5 months/female	US: heterogeneous echogenicity of the mass.	Intestinal obstruction.	Mesentery of ileum. 25 cm from the ileocecal junction.
	1 year/male	ND.	Intestinal obstruction.	Mesentery of ileum.
(24)	4 months/male.	CT scan: showed a large nodular mass in the mesenteric region, displacing bowel loops anteriorly and kidneys and aorta posteriorly. Multiple regions had deposits like perineum and scrotum, omentum, liver, perisplenic region, paracolic gutter, pelvis, and on spermatic cords. The tumor showed fibrotic contents centrally. All these findings indicated metastasis of IMT.	Hemangioma and fibromatosis.	Mesentery of colon.
(25)	28 years/female.	US: 6 cm hypoechoic mass with sharp margins. CT scan: 73*61 mm inhomogeneous round tumor with sharp margins with hypodense fatty components in combination with enhancing soft tissues. PET scan: reactive paratracheal lymph nodes.	Differential diagnosis: IMT, liposarcoma, and teratoma.	Small bowel mesentery.
(26)	34 years/male	CT scan: 4.1*5.1*4.5 cm isodense mesenteric mass caudal to the transverse duodenum and anterior to inferior vena cava. With adjacent lymphadenopathy and enlarged right iliac lymph nodes. Inflammatory changes extend into the mesentery of the right colon.	Mesenteric mass.	Mesentery of small bowel.
(27)	37 years/male.	CT scan and US: solid mass in the right side of the abdomen.	Malignant mass.	Mesentery of ileo-jejunal junction.
(28)	67 years/ male.	CT scan of abdomen and pelvis: hypodense, inflamed and tubular-shaped structure in the distal small bowel mesentery associated with free fluid.	Meckel’s diverticulitis.	Mesentery of ileum.
(29)	19 years/female	US: 19*17*11 cm solid mass in pelvic cavity and medium amounts of ascitic fluid.	Solid mass.	Mesentery of terminal ileum.
	39 years/male	CT scan: 15*10*8 cm heterogeneous-density lesion in the left pelvic cavity.	ND.	Mesangial region of sigmoid and rectum. “Sigmoid mesocolon.”
(33)	32 years/ male	CT scan and US: Findings indicate portal vein thrombosis. hypoechoic mass in the mesentery. Angiography: absence of portal trunk and presence of collateral vessels.	Portal vein thrombosis associated with cavernomatous transformation.	Mesentery of jejunum.
(34)	7 years/female.	US: showed 10*8*6 cm mass in the right side of abdomen with calcification and vascularity. CT scan: 8.8 × 7.2 × 5.4 cm ill-defined mass, heterogenous, enhancing lesion, mostly neoplastic, arising from mesentery and covering the small bowel.	IMT	Mesentery of ileum.
(35)	5 years/male	US: a mass sized 8*10 cm in the abdominal cavity. It extended from below the pancreas to the pelvic cavity. Inside the mass was a calcified tissue with vascularity.	Differential diagnosis of myofibroblastic tumor and neuroblastoma.	Mesentery of ileum.
(36)	35 years/female	CT scan: soft tissue lesion, heterogenous, attenuating enhancement at right abdomen, and the small bowel was pushed to the left side. Small bowel contrast studies: no mass arises from the small bowel and it was deviated to the left side. These findings reinforce the CT findings of external compression of small bowel by a tumor in the right side.	Gastrointestinal stromal tumor.	Transverse mesocolon and omentum.
(37)	13 years/female	US: hyperechoic mass located in the right lower side of abdomen. CT scan: mass originated from the terminal ileum and dilatation in the proximal intestinal loops.	Burkitt lymphoma.	Mesentery of the ileocecal region.
	15 years/male	US and CT scan: revealed a mass originating from the terminal ileum mesentery.	Lymphoma	Ileocecal mesentery.
(38)	14 months/female	US: partially lobulated hypoechoic mass in umbilical region. CT scan: 10*10 cm solid mass but with unclear origin.	Solid mass.	Mesentery of duodenojejunal junction.
(30)	14 years/female	CT scan: Hypodense, well-delineated retroperitoneal tumor surrounding the superior mesenteric vessels, with enlargement of surrounding lymph nodes.	Unconfirmed.	Mesentery of small bowel.
(31)	28 years/female	CT scan: well-marginated, heterogeneous mass in the small bowel mesentery. It was close to superior mesenteric artery and jejunal branches. Center of the mass has low attenuation. MRI: T1 and T2 signal showed decreased intensity in the center of the mass which interpreted the presence of internal blood products or fibrous tissues.	Differential diagnosis of desmoid tumor and gastro-intestinal stromal tumor.	Mesentery of small bowel.
(32)	8 years/male	US: mixed echogenicity mass and well circumscribed in right upper abdomen. In the next day the mass was in the left upper abdomen. Gallium scan: revealed the mass in the right upper side. CT: revealed the mass in the upper right.	Lymphoma	Transverse mesocolon.
(39)	67 years/female	US: well-defined lobulated solid hypoechoic mass with internal vascularity. CT scan: well-defined, hyperdense mass with hypodense areas. Enhancing lesions.	Malignant gastro-intestinal stromal tumor.	Transverse mesocolon.
(40)	28 years/female	CT scan: 8 cm pelvic mass, non-calcified and showed well-defined margins. Before injection of contrast, it showed homogeneous attenuation, after it showed peripheral enhancement. The center is hypo attenuated. MRI: with gadolinium showed peripheral intense enhancement while the center had fibrotic components and was hypo vascular.	ND.	Mesentery of the terminal ileum.
(41)	38 years/female	CT scan: huge abdominal soft tissue mass with hypodense foci of degeneration. It was displacing adjacent jejunal loops laterally and encasing vessels.	Desmoid tumor.	Mesentery of proximal jejunum.
(42)	4 months/female	CT scan: large abdominal hypodense mass displacing the bowel. No clear tumor invasion.	Mesenteric cyst or teratoma.	Mesentery of ileum.
(43)	35 years/female.	CT scan: well-defined 8.2 cm mass with heterogeneous intense enhancement in the right lower quadrant.	ND.	Mesentery of colon.
(44)	4 months/male	CT scan: cyst-like mass attached to the small intestine in the right abdomen, below the liver, and anterior to the kidneys.	ND.	Mesentery of ileum.
(45)	34 years/female	X-ray: dilated small bowel loops with fluid level.	Intestinal obstruction.	Mesentery of ileoceum.
(46)	73 years/female	CT scan: Heterogenous enhancing and ill-defined mass, measuring 12*9*11 cm. Also, there are multiple non-enhancing lesions and small central intralesional calcifications. No metastasis.	Epithelioid variant of an IMT.	The mesentery of the small bowel.
(47)	9 years/female.	CT scan: 9 cm mass characterized by homogenous pattern, no calcification notes. US: a lobulated, solid mass measures 9 cm and contains some necrotic areas.	ND	The mesentery of the distal ileum.
	7 years/female.	Urogram: 7 cm mass, calcified and in homogenous mass, located supravesically and obstructing the uterus.	Histiocytoma.	Small bowel mesentery, with attachment to the segment of mid-small bowel, rectosigmoid, posterior wall of uterus, right broad ligament, and dome of bladder.
	5 years/male	Pelvic x-ray: 1 cm midline pelvic mass, that is calcified at some areas.	ND.	Mesentery of distal ileum.
(48)	2 years/male	CT scan abdomen: A mass located in RLQ takes the place of the ascending colon.	ND.	Mesentery of small intestine.

**TABLE 3:** Pathological features of the reported cases of inflammatory myofibroblastic tumor of the mesentery.

References #	Pathological features	Immunohistochemistry
Our case	**Gross appearance:** Large tannish glistening tumor mass in the center of the bowel loop, the other parts of the bowel were completely normal with attached pericolic fat that is also normal. **Microscopy features:** Myofibroblastic spindle cells in a loose myxoid stroma with minimal cellular atypia but no significant mitosis. Abundant chronic mononuclear inflammatory cells, mostly lymphocytes ± plasma cells, histiocytes and eosinophils. Lymphoid aggregations were scant.	**+Ve**: SMA. –**Ve:** ALK, CD117, DOG1, and Desmin.
(4)	**Gross appearance:** well-circumscribed, multinodular, grayish-white firm tumor with foci of hemorrhage, necrosis, and cystic change. **Microscopy features:** spindle cell tumor within a collagen-rich vascular stroma, with abundant inflammatory cells. The muscular layer of the bowel wall was invaded. Scattered giant cells and focal increased mitotic activities found.	**+Ve:** SMA and HHF-35. –**Ve:** CD34 and CD117.
(5)	**Gross appearance:** No infiltration to the adjacent structures. **Microscopy features:** spindle cells tumor with fascicular and haphazard growth patterns, mild to moderate pleomorphism and mitosis. Mixed stromal inflammatory cells, including plasmacytes, lymphocytes and few eosinophils.	**+Ve:** ALK-1, SMA and Desmin. **–Ve:** ND
(8)	**Gross appearance:** Firm, polypoid mass with whitish-gray whorled appearance. **Microscopy features:** Interlacing bundles of fibroblastic/myofibroblastic spindle cells with elongated and vacuolated nuclei, in a background of abundant blood vessels and a mixture of stromal inflammatory cells. Sant mitotic figures.	**+Ve:** ALK, Vimentin and SMA. **–Ve:** C-kit, Desmin, CD-34, EMA and CK.
(18)	**Gross appearance:** round mass. **Microscopy features:** the tumor composed of fusiform cells with eosinophilic cytoplasm and elongated or globular nuclei within a fibrotic stroma with vascular congestion and rare inflammatory cell elements of mononuclear WBCs and macrophages. Vascular congestion present. No aneuploidy or hypercellularity.	**+Ve:** Ki67. **–Ve:** BCL2, CD34, CD117, and PS100.
(19)	**Gross appearance:** huge (1,245 g), well-vascularized mass, almost completely calcified/necrotic. **Microscopy features:** No malignant tissues.	ND
(20)	**Gross appearance:** firm, homogenous, grayish white mass. **Microscopy features:** hypocellular smears and fascicles of spindle cells with band nuclei, arranged in a myxoid stroma with focal collagen deposition. Mixed inflammatory cells, including lymphocytes and plasma cells. Benign columnar cells were also noticed.	**+Ve:** ALK, SMA, and Desmin. **–Ve:** ND.
(21)	**Gross appearance:** A 25 cm friable mass at proximal jejunal mesentery, extending proximally to the ligament of Treitz. The texture of the mass was like lymphoma. **Microscopy features:** No jejunal submucosal invasion.	**+Ve:** SMA and Desmin. –**Ve:** ALK-1.
	**Gross appearance:** mesenteric tumor with associated bowel ischemia. **Microscopy features:** spindle and stellate cells tumor settled in a myxoid or hyaline stroma. Admixed with inflammatory cells.	**+Ve:** SMA and Desmin. –**Ve:** ALK-1.
(22)	**Gross appearance:** ND. **Microscopy features:** stellate/spindle cell tumor with epithelioid myofibroblastic cells within a mixed myxoid or compact fibrous stroma. Infrequent mitosis. Plasma cells, lymphocytes and neutrophils were seen.	**+Ve:** ALK-1, ALK-11, and RANBP-2. –**Ve: ND**
(23)	**Gross appearance:** Firm, grayish-white mass. **Microscopy features:** myofibroblasts spindle cells tumor with vascular myxoid stroma and lymphoplasmacytic inflammatory cells infiltrate. No mitosis, necrosis or pleomorphism found.	**+Ve:** Vimentin, SMA, and Desmin. –**Ve:** ALK-1.
	**Gross appearance:** large masses. **Microscopy features:** spindle cells tumor with inflammatory cells. No mitosis.	
(24)	**Gross appearance:** ND. **Microscopy features:** well circumscribed mass composed of myofibroblastic, and fibroblastic spindle cells, arranged in fascicles and storiform patterns, and admixed with inflammatory cells including, lymphocytes, plasma cells, eosinophils, and histiocytes. The background had abundant blood vessels. Little mitotic activity. The tumor infiltrated the adjacent colon.	**+Ve:** ALK, SMA, and Desmin. **–Ve:** CD117, CD34, and S100.
(25)	**Gross appearance:** smooth encapsulated outer surface. **Microscopy features:** spindle cells tumor, without atypia, mixed inflammatory cells, including plasma cells, lymphocytes, and eosinophils. Central tumor necrotic debris. No tumor invasion into the colon or jejunum seen.	**+Ve**: Vimentin, Pankeratin, SMA, and Calponin. –**Ve: ND**
(26)	**Gross appearance:** circumscribed, nodular, solid, yellow-tan tumor with focal necrosis. **Microscopy features:** spindle cell tumor without atypia, arranged in myxoid background, along with admixed lymphocytes, plasma cells, and eosinophils. No mitotic figures. The small and large bowel were normal.	**+Ve**: ALK-1 and SMA. –**Ve:** Desmin.
(27)	**Gross appearance:** circumscribed, nodular, firm, grayish-white mass. **Microscopy features:** spindle cell tumor with fascicular growth pattern with collagenized stroma areas. Plasma cells and lymphocytes are found. No mitosis or extension to the adjacent small bowel.	**+Ve:** SMA and Vimentin. –**Ve:** CD34, CD117, S100, Desmin and Ki-67.
(28)	**Gross appearance:** dense, red (hyperemic) mass. The small bowel appeared inflamed but did not look ischemic or dilated. **Microscopy features:** spindle cell tumor with prominent eosinophils, laying in a myxoid stroma. It was invading the muscularis propria of the adjacent bowel.	**+Ve:** SMA, Desmin, and Cytokeratin. **–Ve:** CD117, b-Catenin, and ALK-1.
(29)	**Gross appearance:** solid grayish-yellow tumor with focal hemorrhage and myxomatous appearance. **Microscopy features:** fasciitis-like nodular pattern with myxoid stroma. Neoplastic ganglion-like cells. Erythrocytes extravasation and numerous inflammatory infiltrates, including neutrophils mostly, eosinophils, lymphocytes, and plasmacytes. Mitotic figures present. The tumor invaded the adventitia, muscular layer, and submucosa.	**+Ve:** ALK, CD30, Desmin, SMC, and RANBP2 gene. –**Ve:** CD117, S100, CD21.
(30)	**Gross appearance:** round, solid mass. **Microscopy features:** spindle cell tumor with a dense polymorphic infiltration of mononuclear inflammatory cells.	**+Ve:** Vimentin, SMA, and CD68. **–Ve:** CD34, CD117, and ALK-1.
(31)	**Gross appearance:** ND. **Microscopy features:** bland spindled/stellate cell tumor with mixed with inflammatory cells in a hyaline stroma. Few mitotic figures and minimal atypia.	**+Ve:** SMA and cyclooxygenase-2 (COX-2). **–Ve:** ALK.
(32)	**Gross appearance:** ND. **Microscopy features:** spindle cell tumor with mixed lymphocytes and plasma cells.	ND.
(33)	**Gross appearance:** well-demarcated, encapsulated hard white mass. **Microscopy features:** myofibroblastic cells with mixed foamy histiocytes and mild lymphoblastic infiltration. No neoplastic cells.	**+Ve:** Vimentin, SMA. **–Ve:** Desmin, S-100 protein, keratin, and CD34.
(34)	**Gross appearance:** ND. **Microscopy features:** interlacing fascicles of elongated spindle cells mixed with plasma cells, histiocytes, lymphocytes, and eosinophils.	ND.
(35)	**Gross appearance:** large, sticky mass. **Microscopy features:** spindle cells/Stellate cell tumor with eosinophilic neoplasm in a vascularized myxoid stroma. Abundant plasma cells and lymphocytes. Lymph nodes with reactive hyperplasia.	ND.
(36)	**Gross appearance:** Circumscribed tumor with focal infiltration into the adjacent fat. **Microscopy features:** spindle or epithelioid myofibroblastic tumor in a myxoid or sclerotic stroma with capillary vessels and mixed plasma cells and lymphocytes.	**+Ve:** ALK and SMA. **–Ve:** CD117 and cdk-4.
(37)	**Gross appearance:** solitary lobulated tumor with a whitish gray gritty sensation cut surface due to calcification. **Microscopy features:** fusiform, spindle cell tumor with a myxoid and hyalinized stroma. Increased stromal capillary vasculature and plasmacytes infiltration. Few mitotic figures. No atypical cells.	**+Ve:** SMA and Desmin. **–Ve:** ND.
	**Gross appearance:** rubbery, lobulated tumor with a whitish gray to yellow cut-surface. **Microscopy features:** bland spindle cell tumor, hyaline or myxoid stroma with lymphocytic infiltration. Few mitotic figures. No anaplasia.	**+Ve:** SMA and Desmin. **–Ve:** ND.
(38)	**Gross appearance:** irregular, lobulated, solid mass. **Microscopy features:** ND.	**+Ve:** ALK, CD3, and CD2. –**Ve: ND.**
(39)	**Gross appearance:** well-circumscribed, nodular mass with multi-lobulated, grayish-white cut section. No hemorrhage or necrosis. **Microscopy features:** spindle/stellate cell tumor with haphazard growth pattern, myxoid stroma with a small blood vessels and mixed inflammatory infiltrate, including plasma cells, lymphocytes, and lymphoid follicles.	**+Ve:** SMA. **–Ve:** ALK-1, CD34, CD117, DOG-1, S100, Ki-67.
(40)	**Gross appearance:** encapsulated, well-defined mass with central fibrotic components. **Microscopy features:** spindle cell tumor with inflammatory cells, including lymphocytes. The ileum, appendix, and lymph nodes were normal.	ND.
(41)	**Gross appearance:** ND. **Microscopy features:** spindle cell tumor with sclerotic, variably myxoid stroma, and mixed inflammatory cells, including plasma cells, lymphocytes, histiocytes, and neutrophils. The tumor infiltrated the nearby jejunal loops. No tumor deposits in mesenteric lymph nodes.	**+Ve:** ALK and Vimentin. **–Ve:** CD117.
(42)	**Gross appearance:** soft to firm, encapsulated, lobulated mass with a grayish-white glistening and myxoid cut-section appearance. **Microscopy features:** epithelioid to spindle cell tumor with eosinophilic cytoplasm, in a myxoid background with numerous blood vessels and mixed chronic inflammatory cells, including mature lymphocytes and plasma cells. Variable mitosis. No necrosis. Lymph nodes showed reactive lymphoid hyperplasia.	**+Ve:** ALK, Desmin, SMA, CD68, and CD30. **–Ve:** ND.
(43)	**Gross appearance:** circumscribed, rubbery, yellowish red tumor. **Microscopy features:** mixed spindle and epithelioid cells, in a myxoid stroma with mixed inflammatory cell infiltrates, including lymphocytes, plasma cells and neutrophils. Infrequent mitotic figures.	**+Ve:** ALK-1, SMA, CD30, and RANBP2. –**Ve:** cytokeratin, CK-4 and CD117.
(44)	**Gross appearance:** solid, non-cystic mass. **Microscopy features:** myofibroblastic spindled cell tumor with a fibromyxoid background, and mixture of chronic inflammatory infiltrates, including lymphocytes and plasma cells.	**+Ve:** SMA, ALK, and Desmin. **–Ve:** c-kit, S-100, and CD34.
(45)	**Gross appearance:** firm, multinodular, whorled cut section with myxoid appearance. **Microscopy features:** plump spindle cell tumor with myxoid or vascular growth patterns in a vascular stroma with mixed inflammatory cell infiltrates including, lymphocytes, histiocytes, plasma cells, and lymphoid aggregates. In addition, Stellate and ganglion-like polygonal cells were noticed.	**+Ve:** SMA, ALK-1, p53, Calponin, Desmin, and Ki67. **–Ve:** ND.
(46)	**Gross appearance:** ND. **Microscopy features:** the tumor invades the ascending colon and the terminal ileum.	ND
(47)	**Gross appearance:** well-circumscribed and firm mass. Gross-cut: it has a homogenous to a trabecular pattern. It appears myxoid, with no hemorrhage or necrosis, but there is a cyst. **Microscopy appearance:** the mass contains spindle cells and mononuclear inflammatory cells (lymphocytes, plasma cells). The appearance of spindle cell proliferation varies between interlacing fascicular pattern and storiform pattern. No mitosis.	ND.
(48)	**Gross appearance:** firm and circumscribed mass. Cut surface: it composed of yellow and white **patches.** **Microscopic** features: haphazard proliferation of fibroblast, lymphocytes, plasma cells, and eosinophils. It has a fusiform pattern and has infiltrated the muscularis propria of the small intestine. Few mitotic figures.	ND

### Clinical presentation of inflammatory myofibroblastic tumor

The mean age for the diagnosis of mesenteric IMT was 22.9 years (range: 0–73 years), and the median was 17 years old. The prevalence of IMT was 55.9% in males and 44.1% in females (male:female ratio = 1.27:1). Apparently, mesenteric IMT has a slight propensity to affect younger males than females. In the present case, the patient was 61-year-old male; hence, age was one of the rare characteristics of his clinical presentation.

Furthermore, we analyzed the most common clinical presentations of mesenteric IMT. A total of 24 patients presented with a palpable abdominal lump (67%, including the present case), 20 patients presented with abdominal pain (56%, including the present case), and 6 patients presented with elevated inflammatory markers (16.7%, including the present case). In some cases, there were several atypical presentations such as diarrhea (*n* = 3), difficult urination (*n* = 1), and obstipation (*n* = 1). In these cases, IMT developed as polyps in the mesentery of the small bowel, sigmoid colon, jejunum, and ileum, respectively. A previously published literature review demonstrated that patients with mesenteric IMT had various complaints. Notably, abdominal pain accounted for 33% of the clinical presentation, followed by a painless palpable abdominal lump. Occasionally, systemic symptoms might accompany those complaints, such as fever of unknown origin, weight loss, malaise, uveitis, anemia, and growth failure in children. Laboratory tests demonstrated abnormalities such as elevated ESR, hypergammaglobulinemia, and thrombocytosis (12).

### Radiological features of inflammatory myofibroblastic tumor

There are no specific radiological features that discern mesenteric IMT from other tumors. The plain abdominal radiograph displays diffuse haziness of mesenteric IMT, and the barium studies show the displacement of the bowel due to the mass effect with possible narrowing of its lumen (12). Routinely, the initial diagnostic tool is the abdominal US. US can differentiate between ill- and well-defined tumor edges. Doppler US confirms an increased vascularity of mesenteric IMT (58). A CT scan with contrast exhibits various enhancement patterns by the mesenteric IMT mass ranging from non-enhancement, peripheral enhancement, and central enhancement. This variability reflects the extent of the fibrotic component of the lesion that delays the enhancement of the contrast (58). In addition, it defines the pattern of the lesion (heterogenous or homogenous), the anatomical site, the shape, and the adjacent structures. Magnetic resonance imaging (MRI) shows mesenteric IMT with a low signal intensity due to its fibrotic component. Fluorodeoxyglucose-positron emission tomography (FDG-PET) scan demonstrates various powers reflecting the complex cellular nature and biological behavior of mesenteric IMT (12, 59).

However, the most common radiological pattern was the heterogeneous pattern accounting for 33% of mesenteric IMT (e.g., our case). Only Diop et al. previously reported a homogeneous pattern by the US for mesenteric IMT (18). Mesenteric IMT often had a well-defined edge (19% of cases, including our case). Other documented characters varied between ill-defined, sharp, and irregular edges. Mesenteric IMT displaces the nearby organ (12). In 8% of the reported cases of mesenteric IMT, the tumor was extremely close to the superior mesenteric artery preventing a complete resection. Notably, 36% of the reported cases presented with hypoechoic (solid) mass on US, while few presented with hyperechoic mass. MRI with contrast showed good enhancement (*n* = 5) and mild enhancement (*n* = 2, including our case).

Two cases demonstrated low central but good peripheral enhancement of the tumor. Notably, 15% of cases emphasized the vascularity of this tumor. Infrequent features included a calcified thickened wall (19).

In conclusion, mesenteric IMT is a heterogeneous vascular hypoechoic mass. It has a well-defined edge and often causes displacement, but it may cause invasion in exceptional cases. Occasionally, IMT could be a distinctive lesion based on its intense enhancement.

### Macroscopic features of inflammatory myofibroblastic tumor

It is extremely hard to have a definite pre-operative diagnosis of mesenteric IMT unless FNAC could be performed (20). Mesenteric IMT develops in both small and large bowel. The mesentery of the ileum (36%, *n* = 13) followed by the jejunal mesentery (17%; *n* = 6, including our case) was the most common locations. Only two patients developed mesenteric IMT in the ileo-jejunal junction. In 25% (*n* = 9) of mesenteric IMT cases, the location was in the small bowel mesentery without specific identification. The most common site in the colon was the transverse mesocolon (11%), while it rarely developed in the mesentery of the sigmoid colon.

Mesenteric IMT has various macroscopic features. They can be sessile or polypoid. They are unique with their white-to-yellow color and firm, fleshy, gelatinous, and whorled appearance. In the cut-sections, secondary changes may be present including hemorrhage, calcification, ulceration, necrosis, and ossification. A polypoid-shaped mass hides inside the intestinal lumen when mesenteric IMT seizes invasion power to penetrate the intestinal wall. Tumors present as a solitary mass ranging in size between 1 and 20 cm. Mesenteric IMT can present as multiple discrete lesions (12, 60, 61). In our literature review, the size of mesenteric IMT ranged from 0.5 to 20 cm. The largest mesenteric IMT size settled in the ileocecal mesentery. The median size tumor was 10 cm, and the mean was 10.8 cm. Occasionally, mesenteric IMT lesions presented as multicentric or multifocal masses [*n* = 5; Mazotas et al. (21), Ma et al. (22), Banerjee et al. (23), Kumar et al. (24)] and not a solitary mass. A total of eight (22%) cases presented as a well-circumscribed tumor, and four cases were encapsulated. Mesenteric IMT could have various surface contours such as lobulated (*n* = 4), nodular (*n* = 4), round (*n* = 4), multinodular (*n* = 2), and irregular (*n* = 1). Mesenteric IMT may have unusual consistencies such as rubbery (*n* = 2) or hard (*n* = 1). One case encountered a polypoid mass where mesenteric IMT was in the ileal mesentery (8). Mesenteric IMT appearance varied between myxomatous (*n* = 2), glistening (*n* = 1), and hyperemic (*n* = 1). Mesenteric IMT can block the blood supply of the attached bowel and causes ischemic (*n* = 4). Groenveld et al. (25), Choi et al. (26), and Koyuncuer et al. (27) reported no involvement of the attached bowel, while four cases reported partial involvement nearby bowel. For example, Mazotas et al. (21) documented the invasion of jejunal serosa, Papanikolas et al. (28) noticed the invasion of the muscular wall of the ileum, Chen et al. (6) reported the involvement of the muscular wall of the jejunum, and Telugu et al. (62) notified that the tumor embraced adventitia, muscular layer, and submucosa of the surrounding bowel.

We concluded that mesenteric IMT could be a well-circumscribed tumor with a myxomatous appearance. It presents diverse surface contours, but round, lobular, and nodular surfaces are the most repeated. Rarely, it may cause ischemia of the bowel.

### Microscopic features of inflammatory myofibroblastic tumor

Although IMTs share histopathological similarities, certain histological differences may determine the tumor’s local growth, metastasis, and recurrence rate (63). IMT has three distinctive pathological presentations, namely, cellular, mixed, and fibrous types. The most common and conspicuous pathological findings are spindle-shaped cells and inflammatory cells including plasma cells and lymphocytes. Three histological patterns exist, of which the most common one is the fibromyxoid pattern representing fasciitis-like changes and containing myxoid and inflamed stroma. The proliferating pattern displays multiplying spindle cells sorted in a storiform or fascicular growth pattern accompanied by two processes, namely, inflammation and mitosis. Usually, this pattern is seen only in tumors with atypical features. The sclerosing pattern represents sclerosed desmoid-like areas that interfere with calcification. Undesirable prognosis results from high mitotic tendency, hypercellularity, and proliferation capacity, all of which are the characteristics of the proliferating pattern (64). Moreover, devastating prognostic characteristics include the presence of bizarrely shaped and round cells instead of spindle-shaped cells, nuclear pleomorphism, and infiltrating borders (12, 62).

Spindle-shaped myofibroblasts were the most evident cell type in mesenteric IMT (87%). Satellite and epithelioid myofibroblasts were also prominent. In all cases, lymphocytes, plasma cells, and occasionally neutrophils, histiocytes, macrophages, eosinophils, and monocytes were present. Giant cells (6), ganglion-shaped tumor cells (29), and cells with elongated and globular nuclei (65) were significantly correlated with the recurrence of IMT. Furthermore, pleomorphism contributed to IMT recurrence (5). Two patients died following the recurrence of mesenteric IMT; in both cases, ganglion-shaped tumor cells were present. Although mitosis was evident in 9, the tumor recurrence rate was only 44%. This may reflect that the extent of mitosis and not mitosis *per se* could determine the recurrence of IMT. The most evident histological pattern was the fibromyxoid/vascular pattern (77%). Only three patients with fibromyxoid patterns had a recurrence. Three cases (9.6%) were reported with fascicular/storiform growth-like patterns. Out of which, only one patient had recurrent tumors later when pleomorphism existed as well. Two patients presented with a sclerosing pattern without recurrent tumor. We concluded that the pathological features favoring a bad prognosis of mesenteric IMT include, but may be not limited to, pleomorphism, mitosis, ganglion-shaped tumor cells, fascicular/storiform growth-like pattern, and the presence of cells with elongated and globular nuclei.

### Immunohistochemistry of inflammatory myofibroblastic tumor

The immunohistochemistry evaluation of mesenteric IMT appeared to be an equivocal approach. The assessment included smooth muscle actin (SMA), muscle-specific actin, ALK protein, desmin, calponin, cytokeratin, vimentin, factor VIII A, CD65, and CD117. Positive reaction to cytokeratin and desmin was different. Anaplastic large-cell lymphoma strongly resembles IMT in the presence of ALK (17). In contrast, the ALK gene discerns IMT from other spindle cell neoplastic tumors like the GIST, inflammatory leiomyoma, and congenital fibrosarcoma. ALK protein is present more often at a young age than in the elderly. ALK plays a vital role in the prognosis and diagnosis of IMT. ALK-positive mesenteric IMT had a better prognosis and a lower rate of recurrence (20).

Herein, we analyzed the results of the immunohistochemistry evaluation of mesenteric IMT. Five cases were excluded since no immunohistochemistry data were present. The most evident marker was the SMA (83.8%, *n* = 31, including our case). ALK protein and desmin were equally represented (48.3%, *n* = 15). Vimentin was present in 8 (25.8%) cases. Mesenteric IMT was inconstantly positive to CD117, DOG1, HHF-35, CD34, Ki-67, S100, CD3, CD2, RANBP-2, CD30, CD21, CK-4, cytokeratin, calponin, pan keratin, epithelial membrane antigen, CDK, COX-2, ki-67, BCL-2, CD-68, and p53. RANBP-2 is associated with unfavorable prognosis and high recurrence rate (67%, *n* = 3). In addition, HHF-35, Ki-67, and CD30 were also linked to unfavorable prognosis and tumor recurrence. In terms of the recurrence of mesenteric IMT, ALK-positive mesenteric IMT was slightly advantageous. Only 26% of the cases reported the recurrence of the tumor. We concluded that the highly assigned markers for mesenteric IMT were SMA, desmin, and ALK. Unfavorable markers with unfavorable prognosis or recurrence of IMT were RANBP-2, HHF-35, Ki-67, and CD30. ALK-positive mesenteric IMT has a lower recurrence rate.

### Surgical treatment of inflammatory myofibroblastic tumor

The treatment of choice for mesenteric IMT is complete surgical resection where other remedies could be considered. Although IMT occasionally tends to be an aggressive tumor, complete excision, chemotherapy, and radiation are the recommended therapy (66–68). The risk of the recurrence of IMT is around 23%. Ill-defined edges and abdominopelvic occupation of the tumor are noticeably attributable factors for this risk (16, 17, 69). Metastatic cases are rare, accounting for less than 5% of our review cases (12). ALK rearrangement is one element that grants IMT a low metastatic feature. Therefore, inhibition of ALK could be a decent therapeutic approach. In fact, ALK-positive tumors have excellent crizotinib (ALK inhibitor) response (62, 70). Corticosteroids achieved a practical effect when used as part of adjuvant therapy and recurrence therapy of IMT (62, 71).

The most effective treatment for mesenteric IMT was complete resection with the attached bowel followed by an end-to-end anastomosis. However, four cases reported the recurrence of the tumor following this approach. Chen et al. reported tumor recurrence for large-sized lesions with unfavorable pathological characteristics and those presenting as two discrete masses rather than one (6). Tumor recurrence for 8 months following a complete resection of the tumor was previously attributed to the multicentric presentation rather than solitary mass (22). The treatment of recurrent IMT was re-excision with a significant bowel length. However, Mittal A. et al. documented that the treatment of recurrent mesenteric IMT with ceritinib showed good responses, which could be due to the positive reaction of the tumor to ALK (72). Other treatment modalities depend on several factors specified to the tumor. Complete surgical resection followed by chemotherapy was reported when tumor pathology was a risk factor for recurrence (12, 30). However, the consequences were unfavored. Other treatment strategies were incomplete surgical resection, NSAIDs, prednisone, and pantoprazole. Those approaches were practical when the fibrotic nature of the mass and adherence to the superior mesenteric artery hinder the complete excision. The use of celecoxib (NSAIDs) gave promising results in COX-2-positive mesenteric IMT (31). In another case, the tumor was partially excised but recurred 3 months postoperative. The patient then received prednisone for 7 days, followed by indomethacin, which gave significant results (18). The use of chemotherapy drugs and diclofenac sodium (NSAID) was also reported to be successful in a case of recurrent mesenteric IMT (30). For the sake of a comprehensive review of the topic, Tables 4–6 summarize the clinical, radiological, and pathological features of nine cases of IMT affecting the colon.

**TABLE 4:** Clinical features of the reported cases of inflammatory myofibroblastic tumor of the colon.

References #	Age/ Gender	Clinical presentation	Location	Treatment	Follow up/Recurrence
(49)	7 years/female	Abdominal pain for 2 months, described by fluctuating in severity. And abdominal distension with hyperactive bowel sounds.	Cecum, with extension to the ascending colon, terminal ileum, ileal mesentery, and mesoappendix.	Surgical resection of the terminal ileum, cecum, and right hemi colon, followed by end-to-end anastomosis.	Follow-up after 2 years revealed no recurrence.
(50)	68 years/male	Abdominal pain, diarrhea, and weight loss. Tender mass in RUQ of abdomen. Anemia, elevated erythrocyte sedimentation rate.	2*3 cm Intraluminal mass inside the colon at hepatic flexure (involving the serosa layer of the colon).	Laparotomy right hemicolectomy followed by ileotransversostomy.	Follow-up after 3 years revealed no recurrence.
(51)	9 years/female.	Lethargy, chest pain, and fever. anemia, elevated inflammatory markers. After 2 weeks of antibiotics for chest infection: fever, abdominal pain, chest pain, weight loss, and constipation. Abdominal mass in RLQ.	13*10*6.5 cm mass in the serosal layer of the cecum, it involves the muscularis propria layer, but the mucosa layer is not involved.	Surgical right hemicolectomy.	ND.
(52)	41 years/female	Abdominal pain and distension associated with vomiting, constipation and diarrhea of 3 months.	Terminal ileum.	Surgical resection and restoration of intestinal transit with termino-terminal ileocolic anastomosis.	Uneventful without complications.
(53)	32 months/male	Pallor, loss of appetite and hypoactivity.	Mid transverse, descending and sigmoid colon.	Surgical resection with end-to-end anastomosis.	Uneventful without complications.
(54)	35 years/male	Anal bleeding after defection for 2 weeks.	Descending colon.	Anterior resection and hand-seeing colocolic anastomosis.	Uneventful without complications.
(55)	30 years/male	Abdominal pain and pyrosis for several weeks.	Below right lobe of the liver.	Partial resection of transverse colon followed by end-to-end anastomosis.	Uneventful without complications.
(56)	30 years/female	Intermittent abdominal pain for 4 months.	Left colon.	Laparoscopic left hemicolectomy.	Uneventful without complications.
(57)	56 years/male	3 months of diffuse abdominal pain, bloody stools, altered bowel habits, loss of appetite and weight, fatigue and flatulence.	Ascending colon.	Right hemicolectomy.	Uneventful without complication, 2 months post-surgery.

**TABLE 5:** Radiological features of the reported cases of inflammatory myofibroblastic tumor of the colon.

References #	Age/Gender	Radiology findings	Preoperative diagnosis	Location
(49)	7 years/female	Abdomen US: demonstrated a large mass in the right lower quadrant, measuring 6 m in diameter.	Malignant neoplasm.	Cecum, with extension to the ascending colon, terminal ileum, ileal mesentery, and mesoappendix.
(50)	68 years/male	CT scan abdomen: rounded-filling defect at hepatic flexure. Barium enema: a mass at hepatic flexure that hinders the passage of enema to the proximal colon.	ND.	2*3 cm Intraluminal mass inside the colon at hepatic flexure (involving the serosa layer of the colon).
(51)	9 years/female	US: an abdominal mass near the bladder. CT scan: mass that has 2 lobules, centrally attenuated proposing necrosis.	DD: Lymphoma, neuroblastome and inflammatory bowel disease. Final diagnosis of IMT achieved by needle-biopsy.	13*10*6.5 cm mass in the serosal layer of the cecum, it involves the muscularis propria layer, but the mucosa layer is not involved.
(52)	41 years/Female	AP abdominal x-ray: showed accentuated distension and dilatation of thin intestinal loops and hydro-aerial levels. Abdominal US: reported concentric thickening of the wall of the ascending and transverse colon with preservation of the visualization of its layers. Abdomen and pelvis tomography: showed invaginated appearance and space-occupying lesion.	Intussusception.	Terminal ileum.
(53)	32 months/male	Lower GI contrast study and CT scan: suggestive of intussusception. U/S not helpful.	Intussusception.	Mid transverse, descending and sigmoid colon.
(54)	35 years/male	CT scan: showed a well-demarcated and homogenous solitary mass in the descending colon. On Contrast enhanced view: the mass had enhanced homogenously in delayed phase.	Inflammatory mass.	Descending colon.
(55)	30 years/male	Upper abdominal U/S: showed a cystic partially solid structure possibly originating from the right kidney. MRI: showed well demarcated solitary mass in upper abdomen located against the liver and right kidney. Contrasted enhanced MRI with gadolinium: administration showed a stained capsule of the mass.	Malignant mass.	Below right lobe of the liver.
(56)	30 years/female	F-FDG PET/CT scan with IV contrast: showed intussusception in the abdomen involving the descending colon.	Pedunculated polyp.	Left colon.
(57)	56 years/male	ND	ND.	Ascending colon.

**TABLE 6:** Pathological features of the reported cases of inflammatory myofibroblastic tumor of the colon.

References #	Pathological features	Immunohistochemistry
(49)	**Gross appearance:** the cut surface of the tumor is tan, firm, trabeculated, and knobby. No necrosis, hemorrhage, or cyst. **Microscopy features:** the tissues have interlacing fascicles of elongated spindle cells and fusiform or stellate myxoid cells mixed with plasma cells, histiocytes, lymphocytes, and eosinophils. There is scarce mitosis.	+Ve: CD20, CD30, CD68, V-III, vimentin, SMA, and desmin. –Ve: cytokeratin and S-100.
(50)	**Gross appearance:** polypoid shape, ulcerated mucosa of the tumor. It has a yellow-gray color and rubbery feeling. **Microscopy features:** the lesion composed of spindle cells mixed with thick blood vessels, plasma cells and lymphocytes.	+Ve: CD34, SMA, CD68
(51)	**Gross appearance:** ND. **Microscopy features:** spindle cells mixed with inflammatory cells.	+Ve: SMA, desmin, cytokeratin, ALK, and S-100. –Ve: CD117.
(52)	**Gross appearance:** polypoid formation of 6 cm of diameter on a pedicle and ulcerated that occupied 85% of the lumen, with smooth and whitish external surface. **Microscopy features:** proliferation of spindle cells and abundant inflammatory infiltrate.	ND.
(53)	**Gross appearance:** ND. **Microscopy features:** extensive surface ulceration and was characterized by a proliferation of plump polygonal or more spindle-shaped cells with amphophilic cytoplasm and vesicular nuclei with prominent nucleoli. The lesion cells were distributed in a patternless fashion within a loose myxoid matrix, which contained numerous thin- walled blood vessels and prominent mixed inflammatory cells, including numerous plasma cells and lymphocytes.	+Ve: S100, keratin AE1/AE3 and desmin. –Ve: smooth muscle actin and anaplastic lymphoma kinase 1.
(54)	**Gross appearance:** 3.9 × 3.8 cm sized mass with fungating surface white to yellow color and invaded muscularis propria. **Microscopy features:** composed of a proliferation of spindle-shaped cells arranged in hyaline material with chronic inflammatory cells, composed mainly of plasma cells and lymphocytes, not neutrophils.	+Ve: smooth muscle actin and vimentin. –Ve: desmin, CD117 (c-kit), anaplastic lymphoma kinase (ALK)-1.
(55)	**Gross appearance:** oval shaped 12 cm diameter tumor with a firm tumor capsule and hemorrhagic with a cystic aspect. **Microscopy features:** dense areas with lymphocytes, plasma cells and history testing embedded in a loose spindle cell-like matrix of fibroblasts, myoblasts and histiocytes. Pseudotumor.	ND.
(56)	**Gross appearance:** polypoid mass (5.0 × 2.9 × 2.4 cm) **Microscopy features:** fibrosis in the central portion of the mass and marked infiltration of inflammatory cells, predominantly eosinophils in the periphery.	ND.
(57)	**Gross appearance:** 20 × 20 × 10 mm sized protruded tumor covered by intact mucosa with a central depressed area. **Microscopy features:** elongated cells with fascicular arrangement admixed with inflammatory cells, predominantly lymphocytes and few plasma cells.	+ Ve: desmin, SMA, perinuclear positivity for ALK, CD34, CD68 and CD117. –Ve: AE1/AE3, S100, B cell lymphoma 2, D99, CD56 chromogranin or synaptophysin and KIT.

## Conclusion

Mesenteric IMT demands vast effort to reveal the diagnosis due to its vagueness in the clinical presentation. Mesenteric IMT resembles each other in plenty of pathological and immunohistochemical characteristics. Unluckily, the final diagnosis is challenging to obtain before getting the sample for the histological report, after the operation, or using FNAC. To the best of our knowledge, the case reported here is the first case of jejunal malignant IMT occurring in an elderly male patient. Radical surgical resection seemed to be curative till the date of reporting.

## Data availability statement

The original contributions presented in this study are included in the article/supplementary material, further inquiries can be directed to the corresponding authors.

## Ethics statement

The studies involving human participants were reviewed and approved by Research and Ethics Committee (REC) College of Medicine and Medical Sciences, Arabian Gulf University. The patients/participants provided their written informed consent to participate in this study. Written informed consent was obtained from the individual(s) for the publication of any potentially identifiable images or data included in this article.

## Author contributions

HA, SAA-S, and RY conceived and designed the study. HA, SAA-S, and NA performed the research process and collected the data. SAA-S performed the statistical analyses. HA, SKA, YN, and SAA-S wrote the original draft of the manuscript. HA, SAA-S, NA, FA-S, and KA-S prepared the figures and tables. HA, SKA, SAA-S, NA, YN, and RY edited and revised the manuscript. HA was the project manager. All authors approved the final version of the manuscript.

## Abbreviations

IMT: inflammatory myofibroblastic tumor
–Ve: negative
+Ve: positive
ND: not documented
SMA: smooth muscle actin
FISH: fluorescence *in situ* hybridization
ALK: anaplastic lymphoma kinase
RANBP1: Ran binding protein 2
NSAIDs: non-steroidal anti-inflammatory drug.
